# Subcellular Peptide Localization in Single Identified Neurons by Capillary Microsampling Mass Spectrometry

**DOI:** 10.1038/s41598-018-29704-z

**Published:** 2018-08-15

**Authors:** Linwen Zhang, Nikkita Khattar, Ildiko Kemenes, Gyorgy Kemenes, Zita Zrinyi, Zsolt Pirger, Akos Vertes

**Affiliations:** 1 Department of Chemistry, The George Washington University, Washington, DC 20052 USA; 2 Sussex Neuroscience, School of Life Sciences, University of Sussex, Brighton, BN1 9QG UK; 3 Department of Experimental Zoology, Balaton Limnological Institute, MTA Center for Ecological Research, 8237 Tihany, Hungary

## Abstract

Single cell mass spectrometry (MS) is uniquely positioned for the sequencing and identification of peptides in rare cells. Small peptides can take on different roles in subcellular compartments. Whereas some peptides serve as neurotransmitters in the cytoplasm, they can also function as transcription factors in the nucleus. Thus, there is a need to analyze the subcellular peptide compositions in identified single cells. Here, we apply capillary microsampling MS with ion mobility separation for the sequencing of peptides in single neurons of the mollusk *Lymnaea stagnalis*, and the analysis of peptide distributions between the cytoplasm and nucleus of identified single neurons that are known to express cardioactive Phe-Met-Arg-Phe amide-like (FMRFamide-like) neuropeptides. Nuclei and cytoplasm of Type 1 and Type 2 F group (Fgp) neurons were analyzed for neuropeptides cleaved from the protein precursors encoded by alternative splicing products of the FMRFamide gene. Relative abundances of nine neuropeptides were determined in the cytoplasm. The nuclei contained six of these peptides at different abundances. Enabled by its relative enrichment in Fgp neurons, a new 28-residue neuropeptide was sequenced by tandem MS.

## Introduction

Within a eukaryotic cell, enzymes, metabolites, and signaling molecules are highly compartmentalized, which is due to their localized production and organelle functionality^1,2^. Some small peptides can play distinct roles in cells and organisms depending on their subcellular locations. For example, a well-known neurotransmitter in mammalian central nervous system (CNS), neuropeptide Y (NPY), packaged in dense core vesicles in the cytoplasm, can be secreted via regulated exocytosis to transduce signals^3^, whereas the NPY in the nucleus might regulate specific gene expression^4^. In the CNS of most animal species, neurons are highly heterogeneous cell populations that produce, store and release specific types of neuropeptides^5,6^. To understand the localized regulatory functions and the related neurodegenerative diseases, subcellular analysis of neuropeptide distributions in individual neurons preferably of known function is necessary^7,8^. Animal models with a relatively simpler nervous system and low number of neurons include the pond snail (*Lymnaea stagnalis*) with several easily distinguishable neurons of known function. For example, the 15–18 F-group (Fgp) neurons are known to participate in the physiological functions of cardioexcitatory effects, penial control, and withdrawal in molluscan species^9,10^. Whereas Phe-Met-Arg-Phe amide-like (FMRFamide-like) neuropeptides have been detected in these cells, their subcellular localization is not fully explored. One of the major challenges for subcellular analysis is to produce organelles with high purity and minimum chemical and mechanical perturbations. Facing these challenges, development of new approaches for analyzing subcellular compartments is necessary.

Cell fractionation by centrifugation has been widely used for isolating organelles in bulk samples followed by molecular analysis^11,12^. However, using this method, cell-to-cell heterogeneity is obscured, and extensive sample preparation steps are required to yield subcellular fractions with high purity. Flow cytometry has been adapted for localization and quantification of specific proteins at a rate of thousands of cells per second^13,14^. With the development of advanced fluorescent probes and optical techniques, the subcellular locations, movements, and amounts of pre-labelled peptides and proteins can be determined and quantified by fluorescence microscopy imaging^15–17^. Immunofluorescence, which employs antibodies with fluorescent labels to target specific epitopes on the molecules of interest, has been used for the visualization of subcellular peptide and protein distributions in fixed cells or tissues^4,18,19^. However, some antibodies are limited in their specificity due to alterations of biochemical composition in live cells induced by sample fixation^20^, false detection of other molecular species containing the targeted epitope, i.e., cross-immunoreactivity^21^, and failing to recognize modified forms of peptides or proteins present in cells^22,23^.

With its high specificity and sensitivity, mass spectrometry (MS) is a reliable technique for identification and quantitation of a broad range of molecules, including metabolites, lipids, peptides and proteins in single cells^24–26^. However, sampling and detection of these chemicals within subcellular structures by MS pose significant challenges, such as the fast molecular turnover rates for some compounds, low sample volumes, limited amounts of analytes and the difficulties of isolating individual compartments with high purity. Only a few MS methods have been used for molecular analysis on the subcellular level. With high spatial resolution, secondary ion MS^27,28^ and matrix-assisted laser desorption ionization (MALDI)-MS^29–32^ have been applied for subcellular imaging of metabolites, lipids, peptides, pharmaceuticals and antibiotics in individual cells under vacuum conditions. A recent study demonstrated the extraction of lipid droplets from single cells followed by matrix deposition for MALDI-MS analysis^33^. The peptide content of dense core vesicles has been studied by capillary microsampling combined with MALDI-MS analysis^34^. A neurotransmitter, D-aspartate, was quantitatively determined in subcellular regions of individual *Aplysia californica* neurons by capillary electrophoresis laser induced fluorescence detection^35^. With the capability to analyze subcellular compartments under ambient conditions, laser ablation electrospray ionization (LAESI) MS^36^, live single cell MS^37–39^, and nanospray MS^40^ have been developed for analyzing metabolites and lipids in cell cytoplasm, nucleus, granules and lipid droplets. Recently, mass cytometry has been adapted for imaging of the pre-labelled proteins by metal tagged antibodies in tumor tissues with subcellular spatial resolution^41^.

The capillary microsampling technique utilizes a pulled capillary with a sharp tip to extract the contents of single cells followed by electrospray ionization (ESI) MS with ion mobility separation (IMS). It has been applied for metabolic and lipidomic analysis of single *Arabidopsis thaliana* (*A*. *thaliana*) epidermal cells^42^ and human hepatocellular carcinoma cells^43^. Using this technique, the presence of distinct metabolic pathways in different *A*. *thaliana* cell types was studied^42^. Cellular heterogeneity among individual hepatocytes in response to xenobiotic treatment and due to passing through specific mitotic stages were also revealed^43,44^. These studies were conducted on the cellular contents without a distinction of particular subcellular regions, and they obscured the compositional variations within a cell.

Here we apply capillary microsampling ESI-IMS-MS for subcellular analysis of single identified Fgp neurons from the *L*. *stagnalis* CNS. Distinct sets of neuropeptide species are detected from the cytoplasm of different Fgp neurons with alternative mRNA splicing of the FMRFamide gene. The neuropeptide levels in the cytoplasm and nucleus of Type 2 Fgp neurons show discernible differences. A 28-residue neuropeptide was found for the first time and sequenced by tandem MS in a single Type 2 Fgp neuron.

Not only do these novel findings in *L*. *stagnalis* demonstrate the feasibility of detecting peptide localization in single identified neurons with sub-cellular resolution but they also open new avenues for the analysis of sub-cellular level changes underpinning memory function and dysfunction in both invertebrate and vertebrate model organisms.

## Results

### Peptides resulting from alternative mRNA splicing in cytoplasm

A microscope image of a freshly prepared *L*. *stagnalis* CNS with the individual ganglia labelled is shown in Supplementary Fig. 1a. Some of the neuron types are identifiable based on their size, color, location, and electrophysiological characterisctics^10,45–47^. In the left lateral region of the visceral ganglion, a cluster of ~15–18 cells with diameters of ~45–105 µm can be identified as the Fgp neurons^48^. Individual Fgp neurons were isolated by a fire-polished capillary with an inner tip diameter of ~150–200 µm and transferred into a drop of saline in a Petri dish (see Supplementary Fig. 1b).

**Figure 1:**
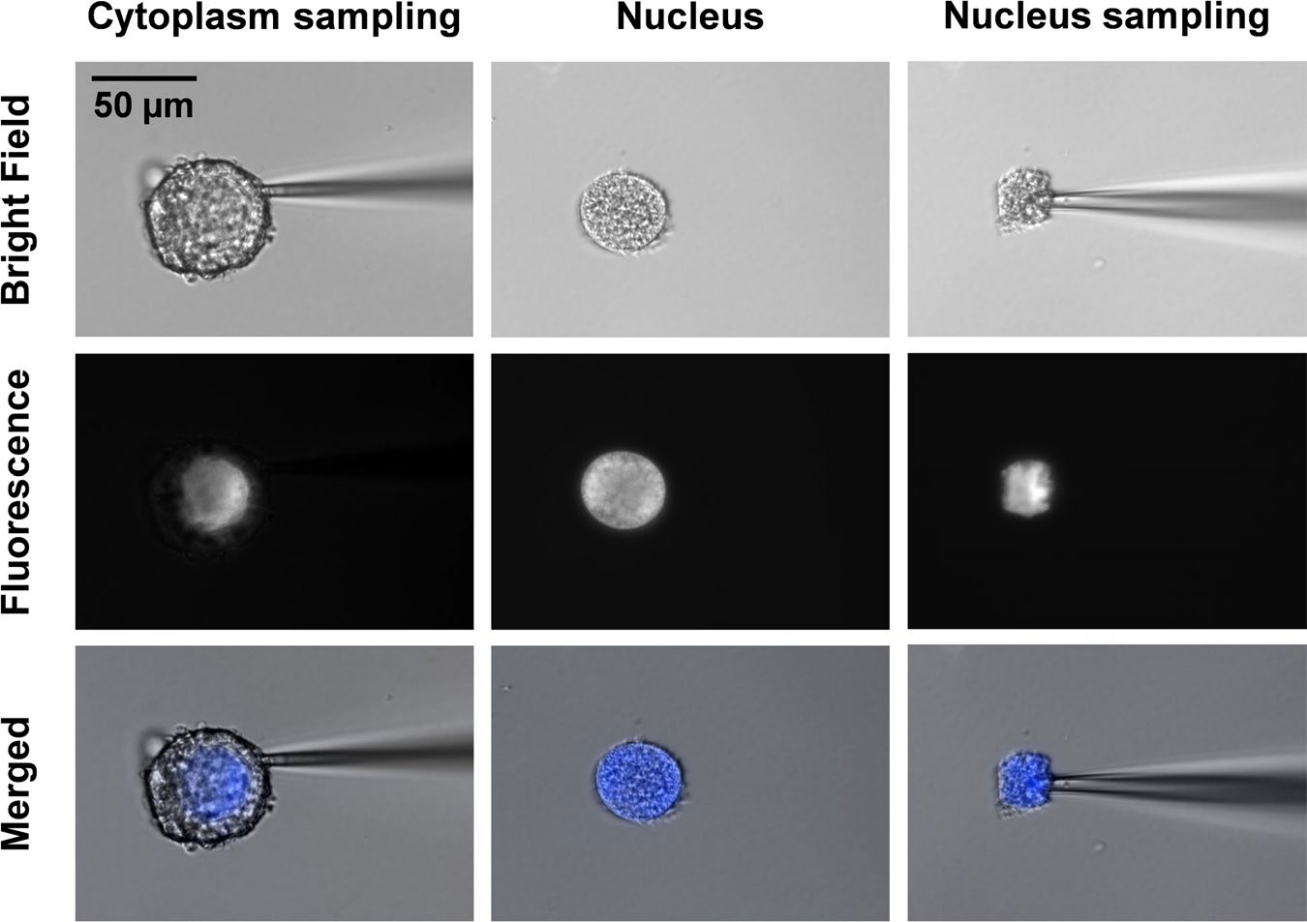
Bright field (top row), fluorescence (middle row) and merged (bottom row) microscope images for subcellular sampling of cytoplasm and nucleus from a single *L*. *stagnalis* Fgp neuron. Scale bar is 50 µm.

To visualize the nucleus, the double stranded DNA (dsDNA) was stained with Hoechst 33342 before the isolation of the single neuron from the ganglion. Figure 1 shows the bright field (top row), fluorescence (middle row), and merged (bottom row) microscope images for subcellular microsampling of a single Fgp neuron. The observed fluorescence signal was associated with the position of the nucleus. A capillary tip with an opening of ~10 µm was used to extract the cytoplasm. After cytoplasm sampling, the nucleus remained intact in shape and morphology. A second capillary was inserted to extract the contents of the nucleus. The sampled volumes and masses were estimated to be ~1.5 pL (1.5 ng) and ~0.4 pL (0.4 ng) from the cytoplasm (total volume ~75 pL) and nucleus (total volume ~25 pL), respectively. On average, ~2% of the total cytoplasm or nucleus volume was extracted and analyzed by capillary microsampling ESI-IMS-MS. As a result, a three-dimensional dataset comprised of ion abundances as a function of drift time (DT) and mass-to-charge ratio (*m/z*) was generated for each subcellular measurement. The corresponding mass spectrum integrated over all DT values was exported for further data processing.

**Figure 2:**
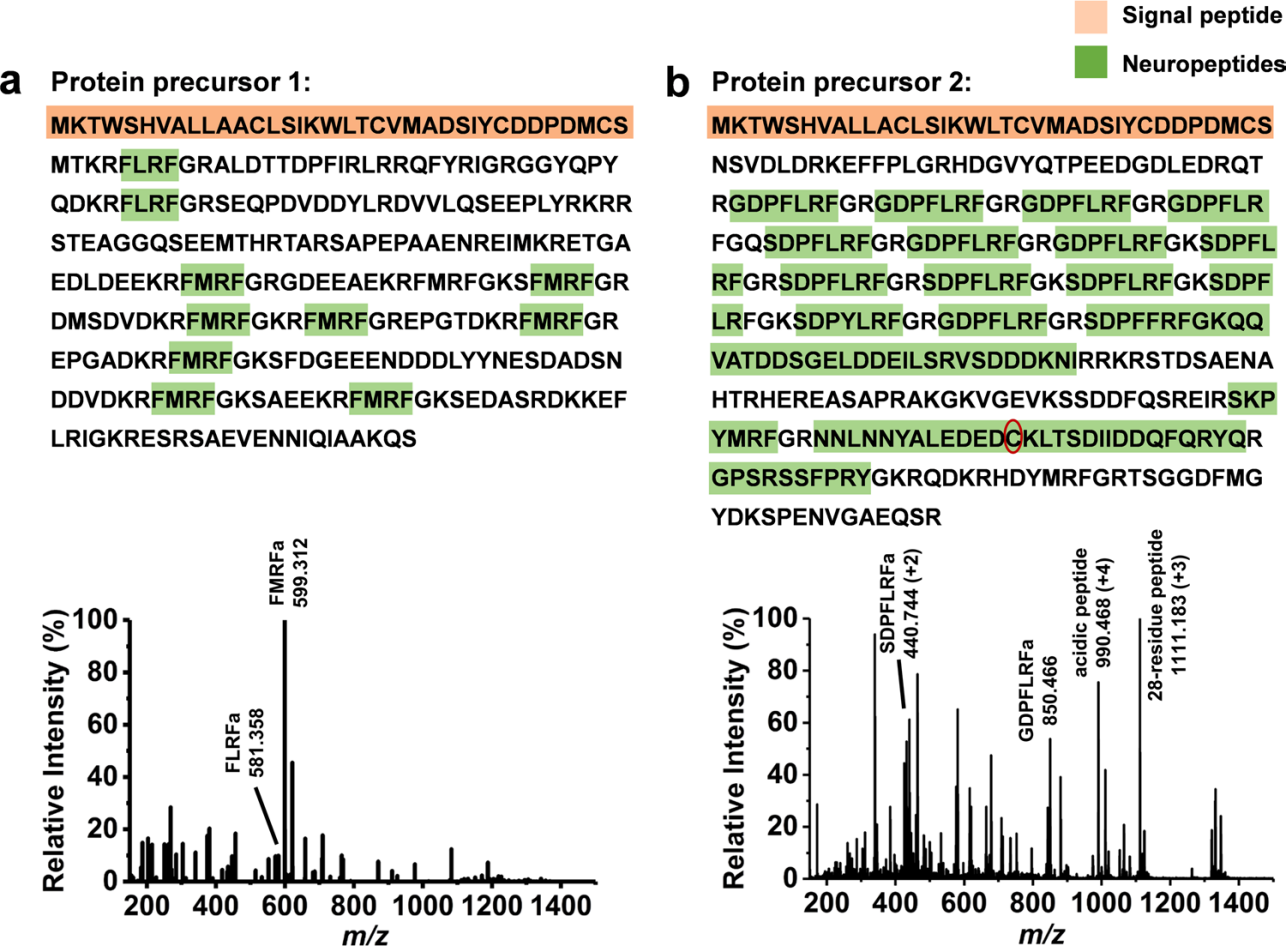
Protein precursor sequences predicted from cDNA analysis for **(a)** Type 1 and **(b)** Type 2 Fgp neurons are shown in top panels. Mass spectra from corresponding cytoplasms are shown in bottom panels of Fig. 2a and b. Signal peptide sequences are highlighted in orange, whereas neuropeptides are marked by green. In Fig. 2b, newly discovered 28-residue neuropeptide is shown in third row from the bottom in the protein precursor 2 sequence, with the cysteine residue marked by red oval conflicting with the glycine residue in our de novo peptide sequence (see Fig. 3).

Previous studies have shown the presence of alternative mRNA splicing of the FMRFamide gene in *L*. *stagnalis* Fgp neurons^49,50^. The spliced mRNA encode two types of protein precursors to produce distinct sets of neuropeptides by post-translational processing, which are mutually exclusively expressed in different Fgp neurons^51^. The two protein precursor sequences, Type 1 and Type 2, predicted by DNA sequencing in previous studies are shown in the top panels of Fig. 2a,b, respectively, with the corresponding signal peptides marked by orange color^52,53^. The positive ion mode mass spectra obtained from the cytoplasm of Type 1 and Type 2 Fgp neurons are shown in the bottom panels of Fig. 2a,b, respectively. In the cytoplasm spectrum of Type 1 Fgp neurons, two tetrapeptides, FLRFamide and FMRFamide are identified (see Supplementary Table 1). In the cytoplasm spectrum of Type 2 Fgp neurons, nine neuropeptides, including five heptapeptides, are detected and identified (see Supplementary Table 2). The limited volume of subcellular compartments, and the viscosity of the nucleus samples present significant challenges for neuropeptide detection and identification. Most of the identified neuropeptide sequences are consistent with those predicted from previous cDNA analysis, marked by green in the top panels of Fig. 2a and b ^52,53^. Collision cross section (CCS) values for these neuropeptides are measured by ion mobility separation and reported for the first time (see Supplementary Tables 1 and 2). These CCS values provide additional information for enhanced molecular identification.

### Single cell tandem MS for neuropeptide identification

The neuropeptide identifications are based on the fragmentation patterns of the peptide ions in single cell tandem ESI-IMS-MS, from MALDI-MS/MS of a visceral ganglion, or nano-liquid chromatography (LC)-MS/MS of peptide extractions from five *L*. *stagnalis* CNS. A previous study showed that only ~340 neurons expressed these FMRFamide-like neuropeptides, which represented 1.5% of all the neurons in the snail CNS^54^. For peptide identification, extracts from five snail CNS are studied by nanoLC-MS/MS, and three of these peptides, GDPFLRFamide, SDPFLRFamide, SDPYLRFamide, are detected and identified. The corresponding nanoLC-MS/MS spectra are shown in Supplementary Fig. 2. MALDI-MS/MS spectra of eight neuropeptides obtained from a visceral ganglion are shown in Supplementary Fig. 3. Two other neuropeptide species, a 35- and a 28-amino acid residue peptide, show low abundances in the MALDI mass spectra.

In contrast, detection and identification of these two neuropeptides is achieved by single cell ESI-IMS-MS/MS. This is due to the lack of signal dilution by the 98.5% of cells devoid of these peptides. Single cell tandem mass spectrum of the ion with *m/z* 990.468 and charge state of +4 assigned as acidic peptide based on fragmentation pattern and predicted neuropeptide sequences from previous cDNA analysis is shown in Supplementary Fig. 4.

**Figure 3:**
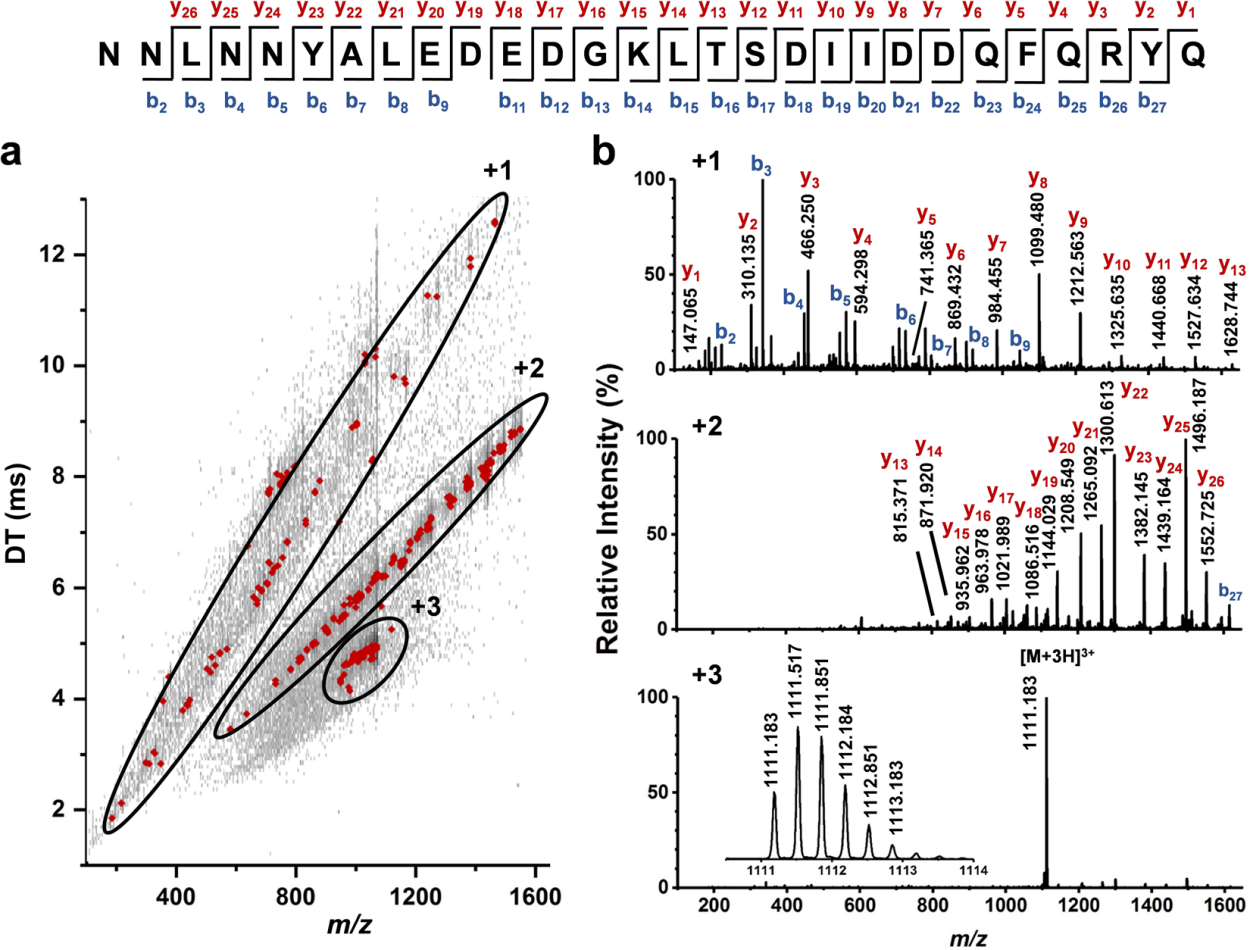
Single cell tandem **(a)** DT vs. *m/z* plot and **(b)** spectra for identification of a peptide with monoisotopic *m/z* 1111.183 and a charge state of +3. This ion is identified as a 28-residue peptide with a de novo sequence of NNLNNYALEDEDGKLTSDIIDDQFQRYQ based on the fragments labelled in the spectra. Fragment and precursor ions in different charge states are separated in distinct mobility regions.

A new neuropeptide with 28-amino acid residues is detected and characterized for the first time from the cytoplasm of a single Type 2 Fgp neuron. For the identification of the 28-residue peptide, the DT vs. *m/z* plot based on the tandem MS of the monoisotopic *m/z* 1111.183 in charge state +3 is shown in Fig. 3a. The precursor ion with a charge state of +3, and the fragment ions with charge states of +2, and +1, are separated in the DT vs. *m/z* plot, and highlighted by distinct ellipses. Figure 3b shows the mass spectra corresponding to the three different charge states with the y- and b-type fragment ions labeled in the top and middle panels. Based on this data, the peptide sequence is identified as NNLNNYALEDEDGKLTSDIIDDQFQRYQ with only a single fragmentation missed at the amino terminus. Surprisingly, the thirteenth amino acid residue in this peptide is identified by tandem MS as glycine instead of the cysteine predicted from cDNA data (see the residue underlined in the sequence above vs. the circled residue in Fig. 2b)^53^. As tandem MS is a direct structural characterization method for identifying the primary structure, we assume that the difference between the codon in the previous literature corresponding to cysteine, and the glycine identified in our study is due to either single nucleotide polymorphism, or a misidentification in the original cDNA data. This discrepancy requires further analysis.

### Neuropeptide distributions between cytoplasm and nucleus

**Figure 4:**
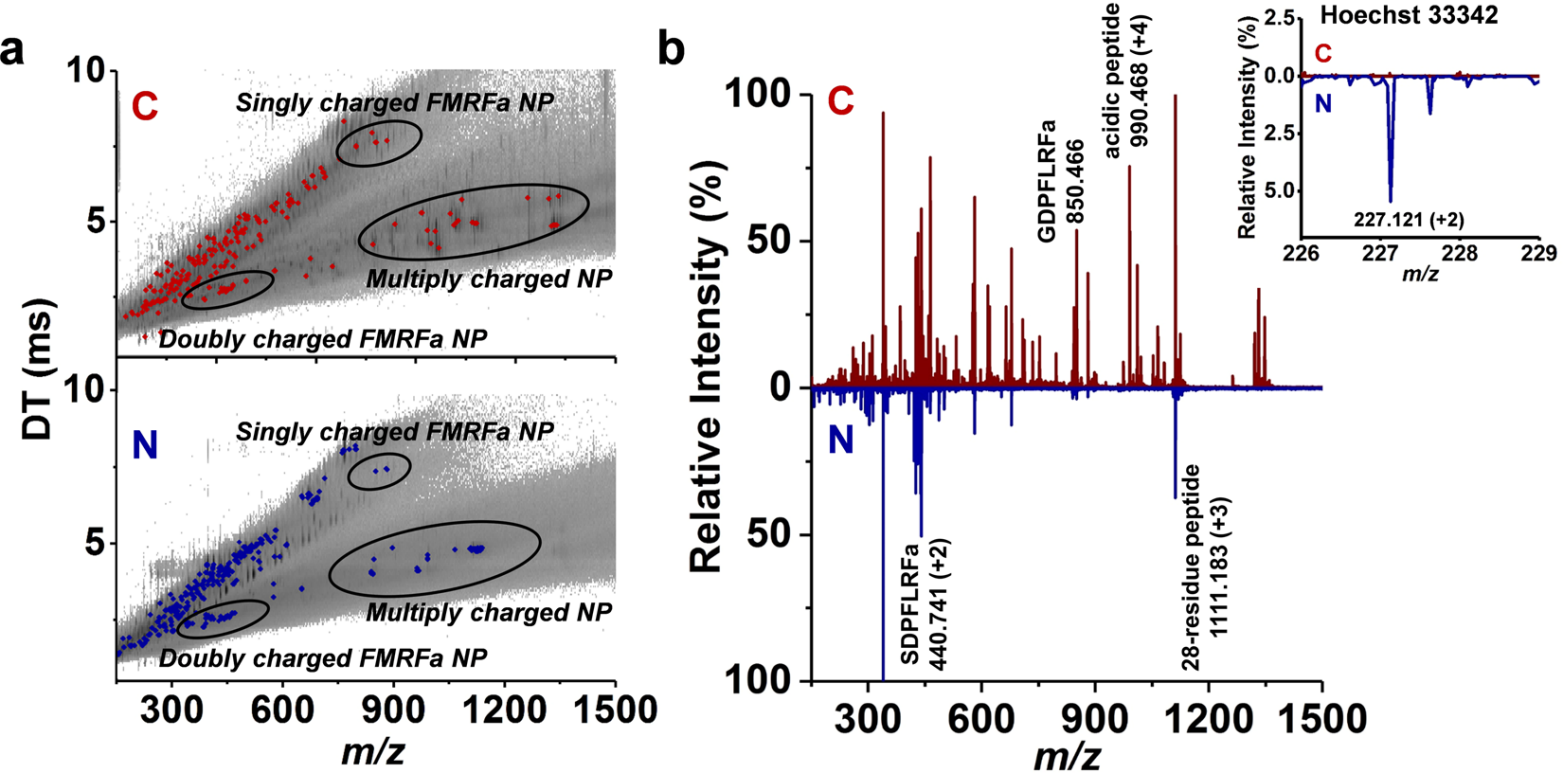
**(a)** Representative DT vs. *m/z* plots, and **(b)** mass spectra for the cytoplasm (C) and the nucleus (N). Inset shows that the *m/z* 227.121 peak for Hoechst 33342 can be detected in the nucleus but not in the cytoplasm indicating successful selective sampling.

To investigate the subcellular distributions of these neuropeptides, the cytoplasm (n = 8) and nucleus (n = 5) of Type 2 neurons were separately sampled and analyzed by capillary microsampling ESI-IMS-MS. In Fig. 4a, representative DT vs. *m/z* plots for cytoplasm (top) and nucleus (bottom) are shown with the singly and multiply charged neuropeptides separated and highlighted in distinct mobility regions. The corresponding mass spectrum for the cytoplasm (top) exhibited peaks corresponding to nine peptides, whereas the spectrum for the nucleus (bottom) only contained peaks from six peptides (see Fig. 4b). Differences in the relative ion abundances between the two subcellular compartments are also observed. The inset in Fig. 4b shows that the ion signal for Hoechst 33342 (*m/z* 227.121) is observed in the nucleus but it is absent in the cytoplasm. This confirms the selectivity of the subcellular sampling process. Due to the lower volumes and higher viscosity of the samples from the nucleus, the absolute abundances and variety of the related ions are relatively low compared to the data from the cytoplasm. It is very challenging to control the extracted volume from cytoplasm and especially the nucleus. Based on all single cell data, ~200 and ~50 molecular features are detected in the cytoplasm and nucleus, respectively. Many of these peaks are related to metabolites and some of them possibly to other, yet unidentified peptides.

**Figure 5:**
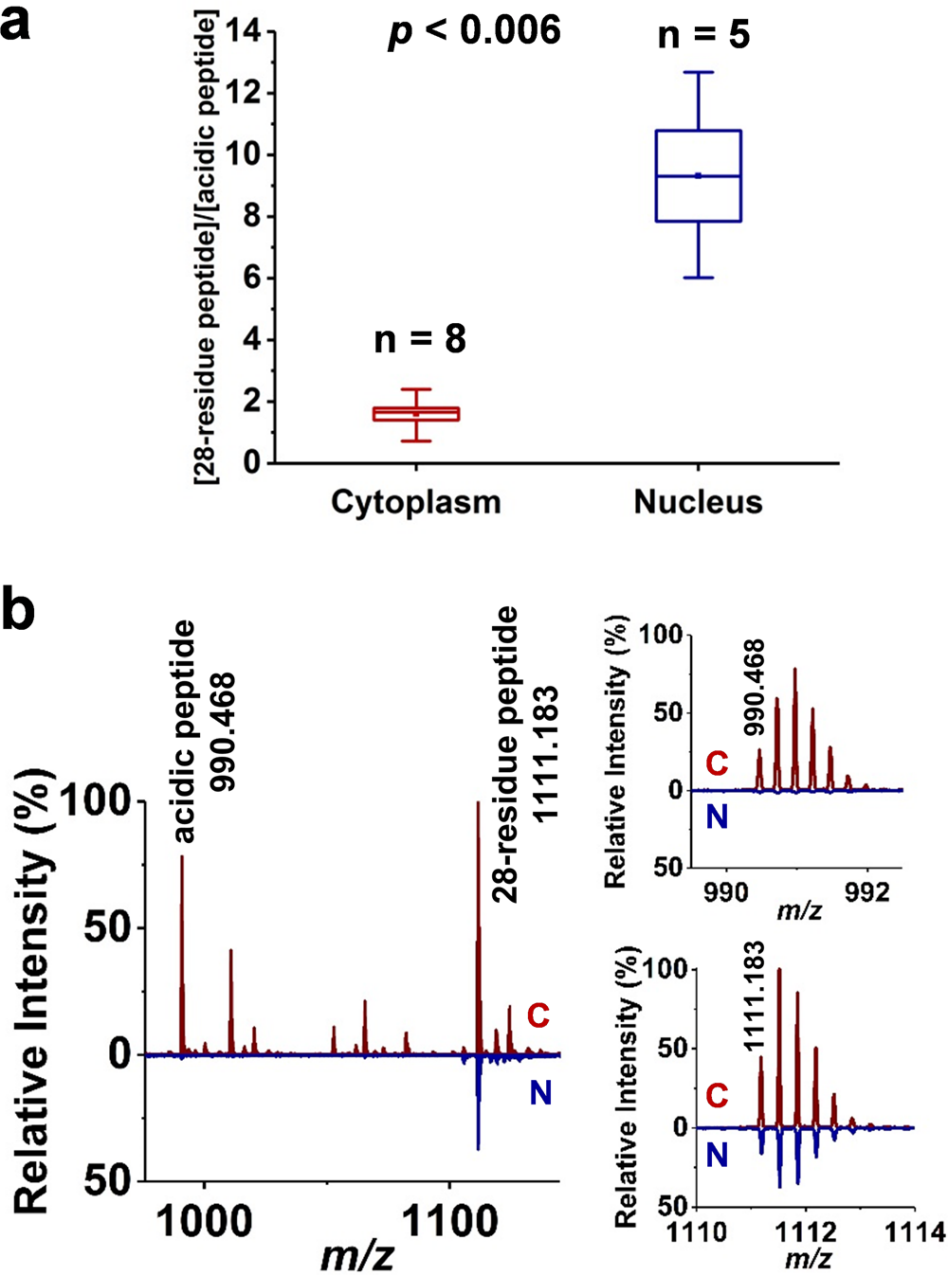
**(a)** Box-and-whisker plot for [28-residue peptide]/[acidic peptide] ratio shows significant difference (*p* < 0.006) between cytoplasm (n = 8) and nucleus (n = 5). **(b)** Mass spectra for acidic peptide (+4, *m/z* 990.468) and 28-residue peptide (+3, *m/z* 1111.183) from cytoplasm (C) and nucleus (N). Acidic peptide shows significantly lower abundances in nucleus compared to cytoplasm.

The normalized abundances of the neuropeptides identified in the cytoplasm (*I*_*C*_) and nucleus (*I*_*N*_) are shown in Supplementary Table 2. The normalization is based on the sum of sample related peak abundances in the spectra. All charge states and adduct forms are accounted for calculating the abundances of specific neuropeptides. Some of the neuropeptides in the cytoplasm, i.e., SDPYLRFamide, SDPFFRFamide, and GPSRSSFPRYamide, are not detected in the nucleus. To find the neuropeptide species with the most variance between the cytoplasm and nucleus, multivariate statistical analysis, in particular orthogonal partial least squares-discriminant analysis (OPLS-DA), was performed. The generated S-plot in Supplementary Fig. 5 highlights the neuropeptides with high correlation and covariance. Our results show that some neuropeptides, i.e., GPSRSSFPRYamide, SKPYMRFamide, FLRFamide, and 35-residue acidic peptide, are the distinguishing features of the cytoplasm spectra, whereas others, i.e., GDPFLRFamide, SDPFLRFamide, PFLRFamide, and Hoechst 33342 dye, are more associated with the spectra from the nucleus. A major difference is found (*p* < 0.006) in the [28-residue peptide]/[acidic peptide] relative abundance ratio that rises from 1.6 ± 0.5 in the cytoplasm to 9.3 ± 3.3 in the nucleus (see Fig. 5a). This is mainly due to the very low abundance of the acidic peptide in the nucleus (see the insets in Fig. 5b).

To obtain additional information on the subcellular distributions of neuropeptides in Fgp neurons, immunostaining of *L*. *stagnalis* CNS by anti-FMRFamide antibody was performed. In Supplementary Fig. 6, confocal microscope images show the presence of FMRFamide (red) only in the cytoplasm (left panel), with the nucleus counterstained by Hoechst 33342 (blue, middle panel). The merged image is shown in the right panel of Supplementary Fig. 6. This observation is consistent with our MS-based results showing FMRFamide neuropeptide present in the cytoplasm of Type 1 Fgp neurons (see Supplementary Table 1).

## Discussion

Most of the neuropeptides are produced in the cytoplasm through the cleavage of larger protein precursors by post-translational processing. For example, the immunostaining images in our study show that FMRFamide is only observed in the cytoplasm of *L*. *stagnalis* Fgp neurons. Peptides can be present in different forms within cell cytoplasm and nucleus. A previous study showed FMRFamide neuropeptide was found to be packaged in dense core vesicles in *A*. *californica* neurons^55^. The antibodies used in immunostaining only recognize the accessible epitopes of these molecules, whereas peptides in their modified forms (e.g., amidated) may not be detected. Other studies showed that due to peptide degradation by peptidases in cells, free peptides were unstable unless binding with proteins or chromatins^56^. The level of free peptides is determined by the relative rate of their production and degradation.

Capillary microsampling MS enables the identification of peptides in subcellular compartments without specifically targeting them. Considering the high abundance of the peptide signal in these cells, it is possible that in addition to the free peptides, this method also detects the peptides released from dense core vesicles^34^ and detached from noncovalent associations with proteins and chromatin during the electrospray process. This untargeted approach opens the door to the detection of localization of multiple peptides (10 in the current study) irrespective of preexisting knowledge on their presence in a cell and the availability of immunoreagents.

Despite the low level of neuropeptides at the tissue level, individual neurons with specific function can exhibit significantly higher concentrations. The high neuropeptide content of these cells enabled the identification of multiple known peptides and the first detection and de novo sequencing of a new 28-residue peptide. Due to the untargeted nature of the MS method, similar findings in other rare cell types can be anticipated even in the presence of posttranslational modifications.

Reports on the detection of peptides in the nucleus are relatively rare in the literature. Immunostaining was used to detect NPY in the nucleus and the nuclear envelope and the authors hypothesized that NPY in the nucleus might play an important role in regulating gene transcription^4^. Large scale separation of nuclei followed by MS has revealed the presence of arginine vasopressin in the nuclei of lymphocytes^57^. However, the mechanisms behind these observations are still not well understood. These neuropeptides can be actively transported or transferred through diffusion into the nucleus^58^.

Finding six peptides in the nucleus of a neuron demonstrates the general utility of the capillary microsampling ESI-IMS-MS approach for subcellular measurements. This is further strengthened by detecting dramatically different peptides in the cytoplasms of Type 1 and Type 2 Fgp neurons. Comparing Supplementary Tables 1 and 2 reveals the presence of two and nine peptides in the cytoplasms of Type1 and Type 2 cells, respectively.

Relative quantitation of subcellular neuropeptide levels is a challenging task. The results demonstrate that capillary microsampling ESI-IMS-MS enables the analysis of subcellular distributions of FMRFamide-like neuropeptides between cytoplasm and nucleus in individual Type 2 Fgp neurons from *L*. *stagnalis* CNS. Relative levels of some neuropeptides show distinct differences between the cytoplasm and nucleus indicating the presence of selective active transport, and/or a difference in production and degradation rates between them. For example, higher levels of the heptapeptides, GDPFLRFamide (*p* < 0.06) and SDPFLRFamide (*p* < 0.09), are present in the nucleus compared to the other neuropeptides. Likewise, the 35-residue acidic peptide is found with significantly higher abundance in the cytoplasm (*p* < 0.01).

The capillary microsampling ESI-IMS-MS approach can be potentially applied for the local analysis of subcellular gradients for a broad range of molecules, such as metabolites, lipids and peptides in diverse cell types. The combination of this method with single cell transcriptomics can serve as a foundation for single-cell systems biology and for subcellular analysis of individual neurons after external stimuli or genetic modification to answer fundamental questions in neuroscience.

## Methods

### Chemicals and materials

HPLC grade methanol and water, and Hoechst 33342 (H1399) were purchased from Thermo Fisher Scientific (Waltham, MA, USA), whereas Instant Ocean sea salt was obtained from Instant Ocean (Blacksburg, VA, USA). Trypsin (T9201), NaCl (S7653), KCl (P9333), CaCl_2_ (C5670), MgCl_2_ (M4880), HEPES (H4034), CaSO_4_ (238988), 2,5-dihydroxybenzoic (DHB, 85707), paraformaldehyde (158127), phosphate buffered saline (PBS, P4417), Dulbecco’s PBS (DPBS, D8537), Triton X-100 (TX, T8787), bovine serum albumin (BSA, A3912), and rabbit anti-FMRFamide primary antibody (AB15348) were purchased from Sigma Aldrich (St. Louis, MO, USA). NorthernLights™ 557-conjugated Anti-Rabbit IgG secondary antibody (red; Cat. No. NL004) was obtained from R&D System (Minneapolis, MN, USA). Snail saline was prepared at final concentrations of 51.3 mM NaCl, 1.7 mM KCl, 4.1 mM CaCl_2_, and 1.5 mM MgCl_2_, and 2 mM HEPES in autoclaved water with pH adjusted to 7.9 by 0.1 M NaOH. The DHB matrix solution was prepared in methanol at a final concentration of 10 mg/ml.

### Sample preparation

Pond snails, *L*. *stagnalis*, were maintained in deionized water (18.2 MΩ·cm) supplemented with 0.26 g/L sea salt and 0.34 g/L CaSO_4_ at room temperature (~20 °C). Snails of ~3–4 months of age and shell length of ~10–15 mm were dissected in saline. Under observation by a stereomicroscope (E-Zoom6, Edmund Optics, Barrington, NJ, USA), the CNS was extracted from the animal by a pair of fine scissors (50086, World Precision Instruments, Sarasota, FL, USA) and pinned out on the bottom of a Sylgard (Sylgard 184, Dow Corning, Midland, MI, USA) dish containing saline (see Supplementary Fig. 1a). After removing the outer sheath membrane by two pairs of fine forceps (500085, World Precision Instruments, Sarasota, FL, USA), the CNS was treated with 0.2% trypsin solution, prepared in saline, for 10 minutes followed by rinsing three times by saline. To stain the dsDNA contained in the nucleus, the CNS was treated for 5 minutes with 1 µg/ml Hoechst 33342 made in saline. After replacing the staining solution with saline, the inner sheath membrane surrounding the visceral ganglion was teared off by the forceps to expose the neurons of interest. A fire-polished capillary with an inner diameter opening of ~150–200 µm was filled with the saline and attached to a micrometer syringe (GS-1100, Gilmont, Gilmer, TX, USA). The capillary was used to isolate individual Fgp neurons from the visceral ganglion by applying gentle negative pressure (see Supplementary Fig. 1a). The isolated Fgp neurons were transferred to a 10 μL drop of saline on a Petri dish by applying positive pressure.

### Subcellular sampling

Initially, glass capillaries (TW100F-3, World Precision Instruments, Sarasota, FL, USA) were pulled by a micropipette puller (P-1000, Sutter Instrument, Novato, CA) installed with a box filament (FB255B, Sutter Instrument, Novato, CA). Sharp capillary tips with long tapering and <1 µm opening were produced. The pulling program was set as Heat = 492, Pull = 60, Velocity = 90, and Delay = 200, at Pressure = 500. Then the capillary tips were cut with a pre-cleaned ceramic scoring wafer (Cat. No. 20116, Restek Corporation, Bellefonte, PA, USA) to generate larger openings with inner diameters of ~10 µm.

A capillary holder (IM-H1, Narishige, Tokyo, Japan) attached with a sampling capillary tip was mounted on a micromanipulator (TransferMan NK2, Eppendorf, Hauppauge, NY, USA) on an inverted microscope (IX71, Olympus, Tokyo, Japan). For sampling the neuron cytoplasm, the capillary tip was gently inserted into the cell without touching the nucleus and a negative pressure was applied through the capillary tip with a syringe attached from the back. After cytoplasm sampling, the nucleus retained its shape and morphology and a second capillary was used to extract its contents. The volumes of the sampled cytoplasm and nucleus were estimated to be ~1.5 pL and ~0.4 pL, respectively. To check for the presence or absence of Hoechst 33342 in the samples, the capillary tips containing the subcellular contents were examined by a fluorescence microscope with a filter cube (49000, Chroma, Bellows Falls, VT, USA).

### Single cell mass spectrometry

The capillary tips containing the sampled subcellular contents (cytoplasm or nucleus) were backfilled with 1.0 µL electrospray solution. The electrospray solution was 50% methanol supplemented with 0.1% acetic acid. The capillaries were inserted in a microelectrode holder (MEW-F10A, Warner Instruments, Hamden, CT, USA) attached with a platinum wire of 200 µm in diameter and ~5 cm in length (Alfa Aesar, Ward Hill, MA, USA). The platinum wire was in contact with the solution to provide electrical connection to the capillary tip. A high positive voltage of +2000 V was applied to the microelectrode by a regulated power supply (PS350, Stanford Research Systems Inc., Sunnyvale, CA, USA) to generate electrospray in front of a quadrupole time-of-flight (TOF) mass spectrometer equipped with a traveling wave (T-wave) ion mobility separation system (Synapt G2-S, Waters Co., Milford, MA, USA).

### Data analysis

A three-dimensional dataset comprised of ion abundances as a function of drift time (DT) and mass-to-charge ratio (*m/z*), i.e., a DT vs. *m/z* plot, was acquired in positive ion mode for each subcellular sample. DriftScope 2.8 (Waters Co., Milford, MA, USA) software was used to process the DT vs. *m/z* plots and derive the ionic CCS values from the measured DTs using singly charged polyalanine oligomer (n = 4–14) mixture as the calibrant. The CCS calibration curve is shown in Supplementary Fig. 7^59^. The mass spectra integrated over all DTs were visualized by MassLynx 4.1 (Waters Co., Milford, MA, USA) and exported to mMass software^60^ for further data processing. The sample related peak lists of *m/z* and ion abundances were generated by mMass through a process of peak picking, deisotoping, and subtraction of the background ions originating from both the electrospray solution and saline, and summing the abundances of all the ionic species for identified molecular features. The processed peak lists for cytoplasm and nucleus spectra were imported into MetaboAnalyst for statistical analysis, in particular, orthogonal partial least squares-discriminant analysis (OPLS-DA) using Pareto scaling^61^.

Neuropeptide identifications were based on a combination of accurate mass measurements and tandem MS by capillary microsampling MS at the single cell level or MALDI-MS on an *L*. *stagnalis* visceral ganglion using DHB as the matrix (see Supplementary Tables 1 and 2). The MALDI tandem MS experiments were performed on a MALDI LTQ Orbitrap XL mass spectrometer (Thermo Scientific, San Jose, CA, USA). The neuropeptide sequences predicted by cDNA analysis in previous studies were compared to our assignments based on tandem MS^52,53^.

### Neuropeptide extraction

Neuropeptide extraction for nanoLC-MS/MS was obtained from five *L*. *stagnalis* CNS and prepared following a previous published protocol with slight modifications^62^. Five *L*. *stagnalis* CNS was prepared in saline and rinsed in cold acidified methanol (90% methanol, 9% acetic acid, and 1% water). The CNS was transferred in a 0.5 mL centrifuge tube and quenched with liquid N_2_. The frozen CNS tissue was grinded by a pestle (Cat. No. 12–141–367, Thermo Fisher Scientific, Waltham, MA, USA) using a pestle motor (Cat. No. 12–141–361, Thermo Fisher Scientific, Waltham, MA, USA). To prevent protein degradation during sample preparation, a protease inhibitor tablet (A32963, Thermo Fisher Scientific, Waltham, MA, USA) dissolved in 80 mL of 20 mM EDTA solution was prepared. The centrifuge tube containing the grinded tissue was added with 50 µL acidified methanol and 50 µL EDTA solution with protease inhibitor. Cell lysis was further performed by sonication using a probe sonicator (Q125, QSONICA, Melville, NY, USA). The sample was centrifuged at 14,000 g at 4 °C for 10 min and the supernatant was transferred in a new tube. To remove larger proteins, the extracted contents were filtered through a 10 kDa MWCO membrane (Cat. No. 88513, Thermo Fisher Scientific (Waltham, MA, USA). The filtrate was dried by a vacuum concentrator (Labconco, Kansas City, MO). The dried sample was reconstituted in 50 µL of 2% acetonitrile solution with 0.1% formic acid for nanoLC-MS/MS injection.

### nanoLC-MS/MS

Peptides separation and identification were performed using a reversed-phase nano-liquid chromatography (model Dionex Ultimate 3000 RSLCnano, Thermo Scientific, Waltham, MA) equipped with a 20 µL injection loop coupled to Orbitrap Fusion Tribrid mass spectrometer (Thermo Scientific, San Jose, CA, USA). Peptides were trapped on a C_18_ trapping column (Cat No. 160454, Thermo Fisher Scientific, Waltham, MA, USA) followed by chromatographic separation using a C_18_ analytical column (75 μm ID, 500 mm length, 3 μm beads with 10 nm pore size, Cat No. 164570, Thermo Fisher Scientific, Waltham, MA, USA). The mobile phases were composed of (A) water with 0.1% formic acid and (B) acetonitrile with 0.1% formic acid. An 85 min gradient was used: 0–10 min, 2% B; 10–10.2 min, 2%-5% B; 10.2–65 min, 5%-80% B; 65–70 min, 80% B; 70–80 min, 80–2% B; 80–85 min, 2% B. Tandem MS data was collected under positive ion data dependent acquisition (DDA) mode using HCD fragmentation at 28% normalized collision energy. Full single-stage (MS^1^) scans were acquired in Orbitrap analyzer at a resolution of 60,000 FWHM, whereas tandem MS scans were collected in the ion trap analyzer.

To document peptide identification, tandem MS raw data and the mzML files from all three instruments (Synapt G2-S, MALDI LTQ Orbitrap XL, and Orbitrap Fusion Tribrid) were uploaded to the PeptideAtlas repository (http://www.peptideatlas.org/). The identifier number for the dataset is PASS01199.

### Immunohistochemistry

*L*. *stagnalis* CNS was dissected and pinned out on a Sylgard dish, and fixed overnight in 4% paraformaldehyde buffered with 0.1 M phosphate buffer (pH 7.4) at 4 °C. The fixed CNS was washed twice in 1 × PBS solution (pH 7.4) for 10 min, followed by incubation overnight in PBS containing 20% sucrose at 4 °C. Cryomicrotome sections of ~16 µm thickness were made and mounted on slides. To block the non-specific binding sites, the slides were washed with PBS containing 0.25% Triton X-100 and 0.25% bovine serum albumin at room temperature. After washing, the slides were incubated overnight at 4 °C with rabbit anti-FMRFamide primary antibody diluted in 1:1000 in PBS-TX-BSA. The slides were further washed twice with PBS-TX for 10 min, followed by incubation for 6 h at 4 °C with the fluorophore-conjugated donkey anti-rabbit IgG secondary antibody at 1:1000 dilution. After washing with modified DPBS, the nuclei were stained with Hoechst (1 μg/mL in DPBS) for 10 min at room temperature. The slides were washed with DPBS and covered with fluorescent mounting medium (Dako Fluorescence Mounting Medium, S3023). The stained tissue sections were analyzed with a Leica DMi8 confocal microscope.

## Electronic supplementary material

Supplementary info
