# Supplementary material for: Subcellular Peptide Localization in Single Identified Neurons by Capillary Microsampling Mass Spectrometry
Source: Sci Rep. 2018 Aug 15;8:12227. doi: 10.1038/s41598-018-29704-z (PMC6093924; doi:10.1038/s41598-018-29704-z)
Supplement: Supplementary file 1 — Supplementary info [file 41598_2018_29704_MOESM1_ESM.pdf]

Supplementary Information for

Subcellular Peptide Localization in Single Identified Neurons by Capillary Microsampling Mass Spectrometry

Linwen Zhang[1], Nikkita Khattar[1], Ildiko Kemenes[2], Gyorgy Kemenes[2], Zita Zrinyi[3], Zsolt Pirger[3], and Akos Vertes[1]*

[1]Department of Chemistry, The George Washington University, Washington, DC 20052, U.S.A.

[2]Sussex Neuroscience, School of Life Sciences, University of Sussex, Brighton, BN1 9QG, UK

[3]Department of Experimental Zoology, Balaton Limnological Institute, MTA Center for Ecological Research, 8237 Tihany, Hungary

*Corresponding Author

E-mail: vertes@gwu.edu. Phone: +1 (202) 994-2717. Fax: +1 (202) 994-5873.

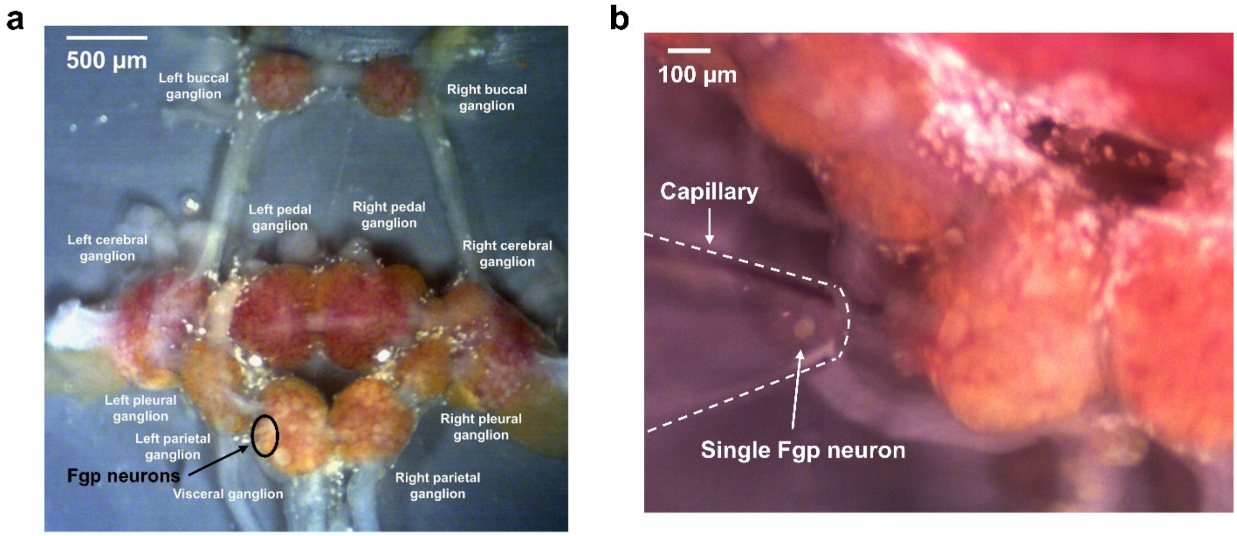

Supplementary Fig. 1. (a) Dissected *L. stagnalis* CNS pinned to bottom of a Sylgard dish with its ganglia labelled. A cluster of Fgp neurons are circled on the left lateral region of the visceral ganglion (scale bar is 500 µm). (b) A single Fgp neuron is isolated by a fire-polished glass capillary (scale bar is 100 µm).

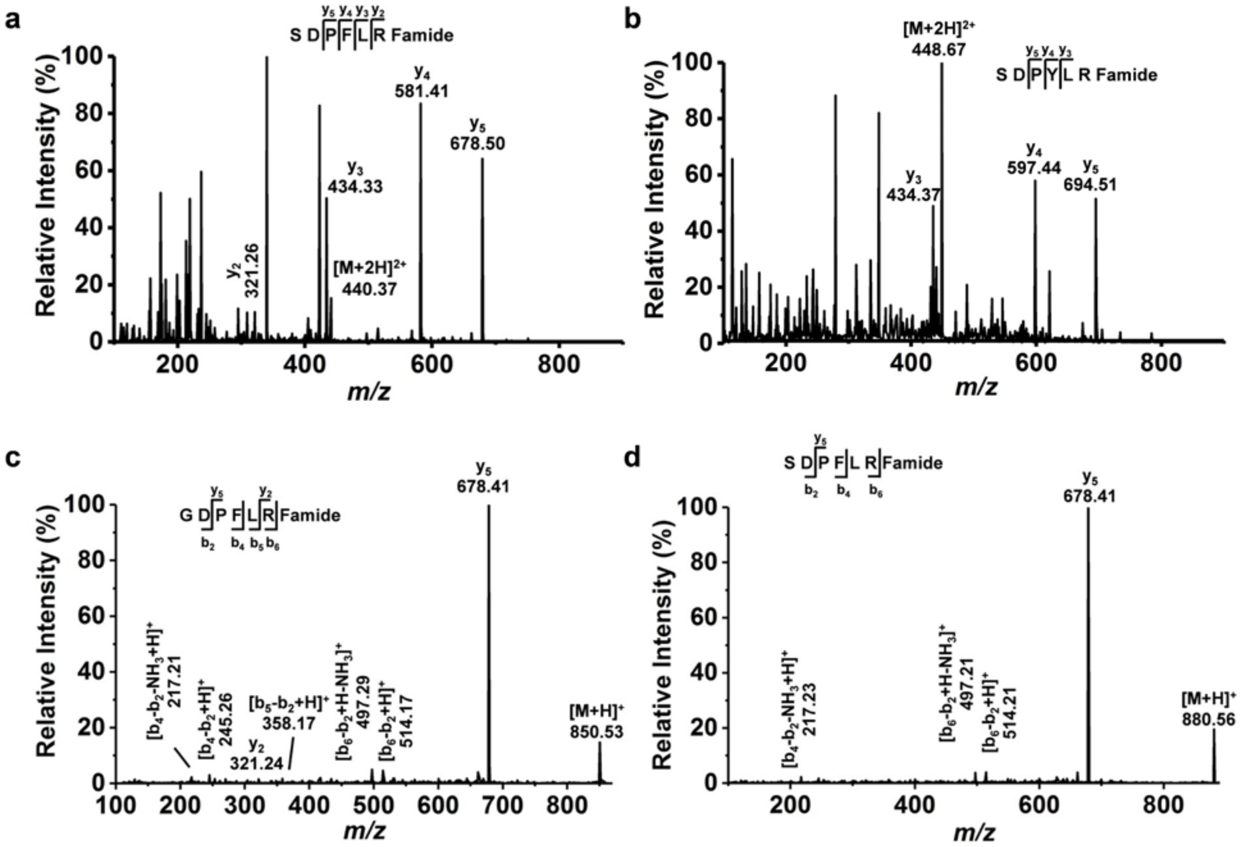

Supplementary Fig. 2. Tandem mass spectra from LC-MS/MS of peptides extracted from five snail CNS.

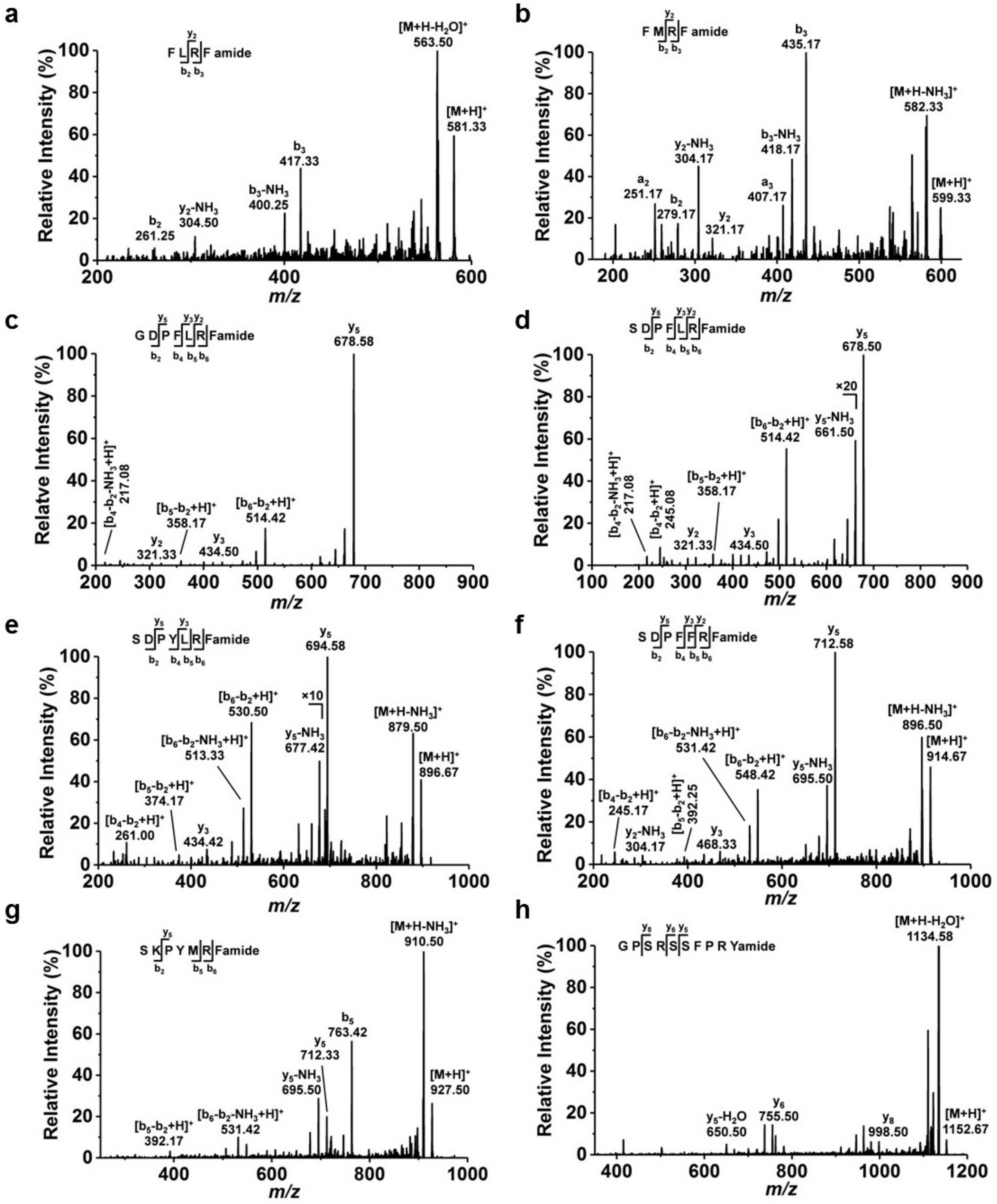

Supplementary Fig. 3. MALDI tandem mass spectra of peptides from a visceral ganglion.

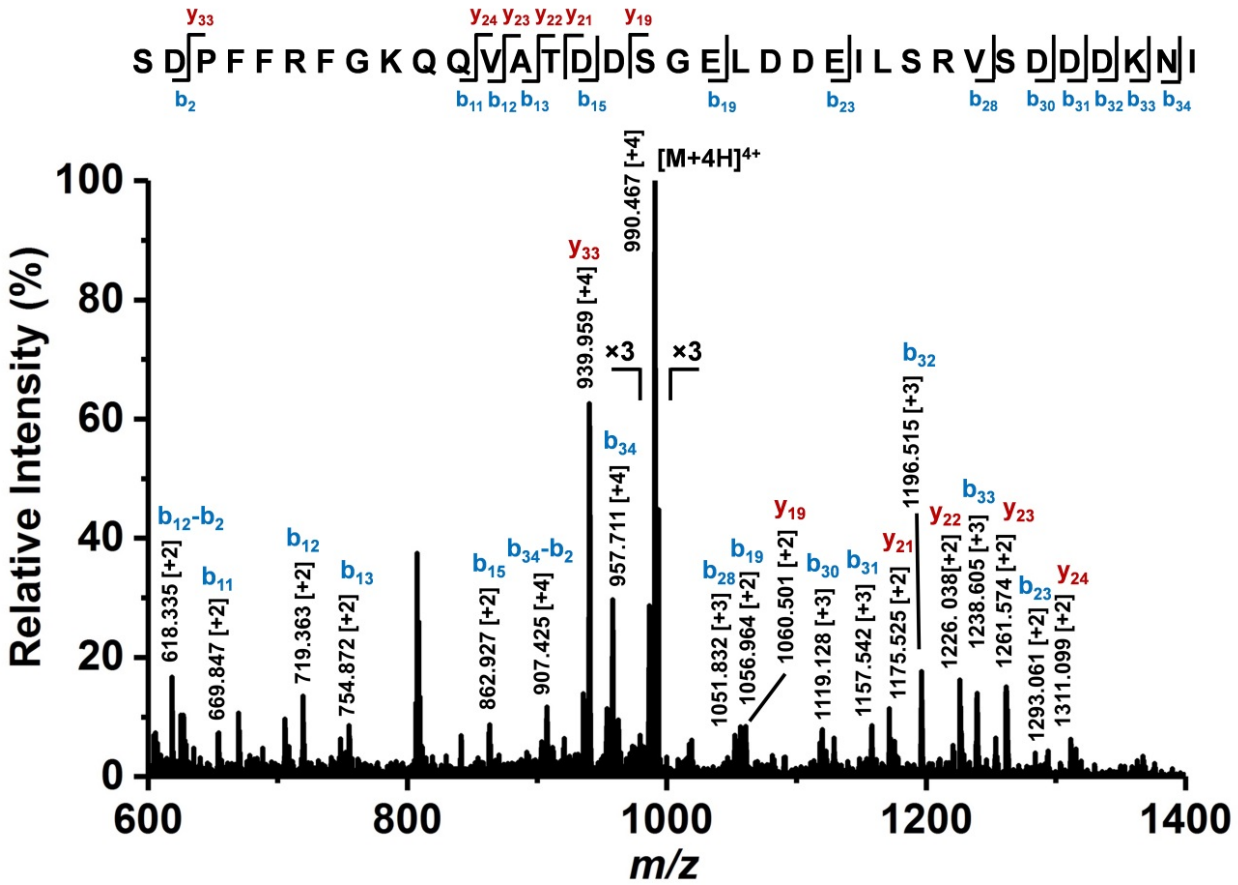

Supplementary Fig. 4. Single cell tandem mass spectrum for a peptide ion with *m/z* 990.468 and a charge state of +4, assigned as acidic peptide, based on fragmentation pattern and predicted peptide sequences.

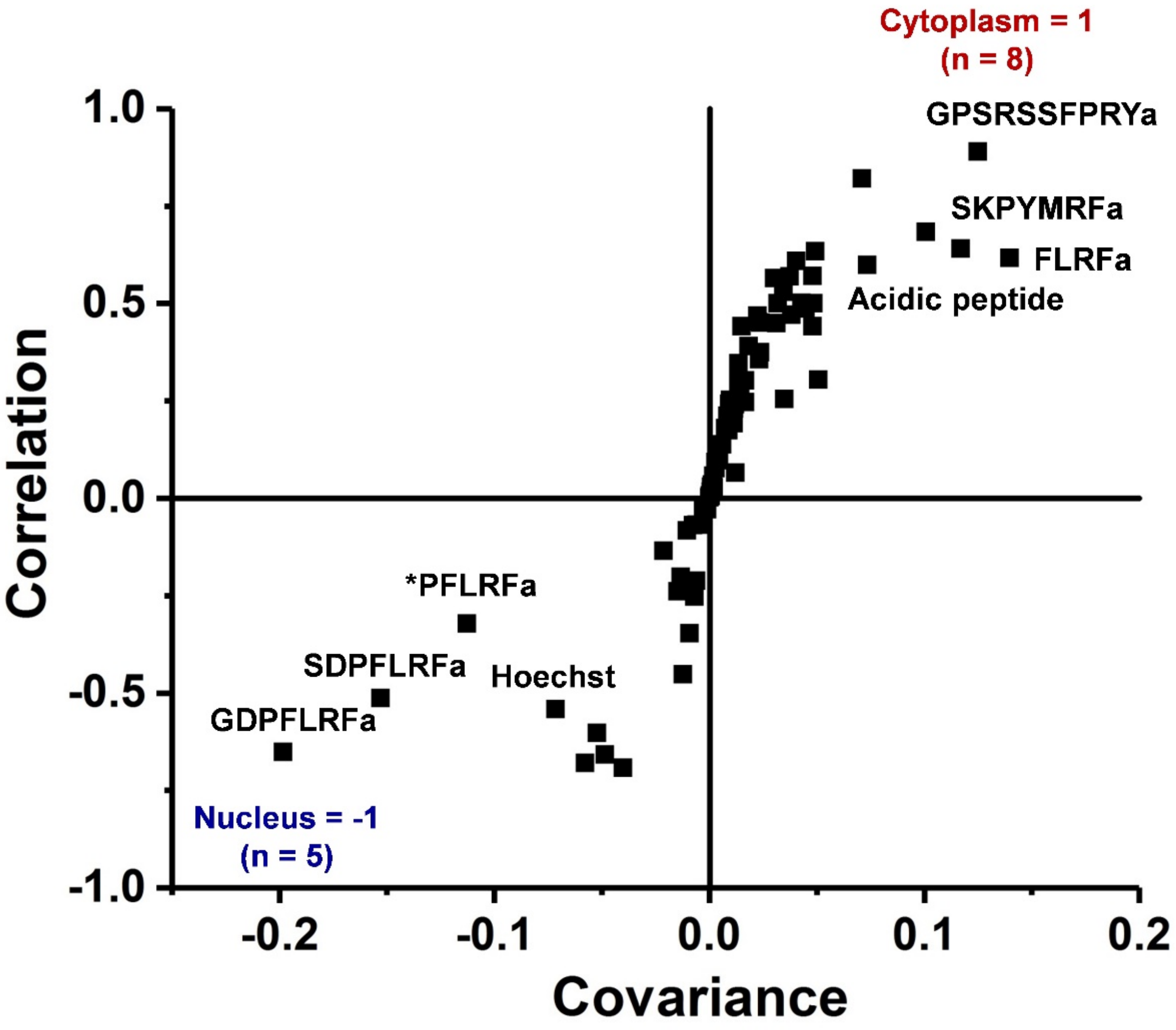

Supplementary Fig. 5. S-plot shows differences in neuropeptide compositions between cytoplasm (n = 8) and nucleus (n = 5). Labelled molecular species on the top right and bottom left quadrants correspond to the neuropeptides with higher abundances in cytoplasm and nucleus, respectively.

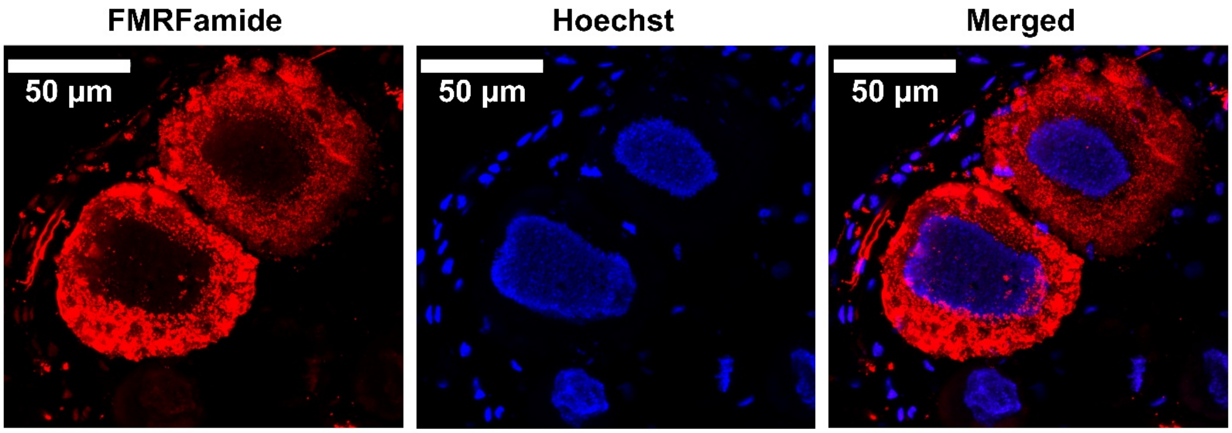

Supplementary Fig. 6. Confocal microscope images for Type 1 *L. stagnalis* Fgp neurons immunostained by anti-FMRFamide antibody in red (left) and counterstained by Hoechst 33342 in blue (middle). The corresponding merged image is shown in the right panel.

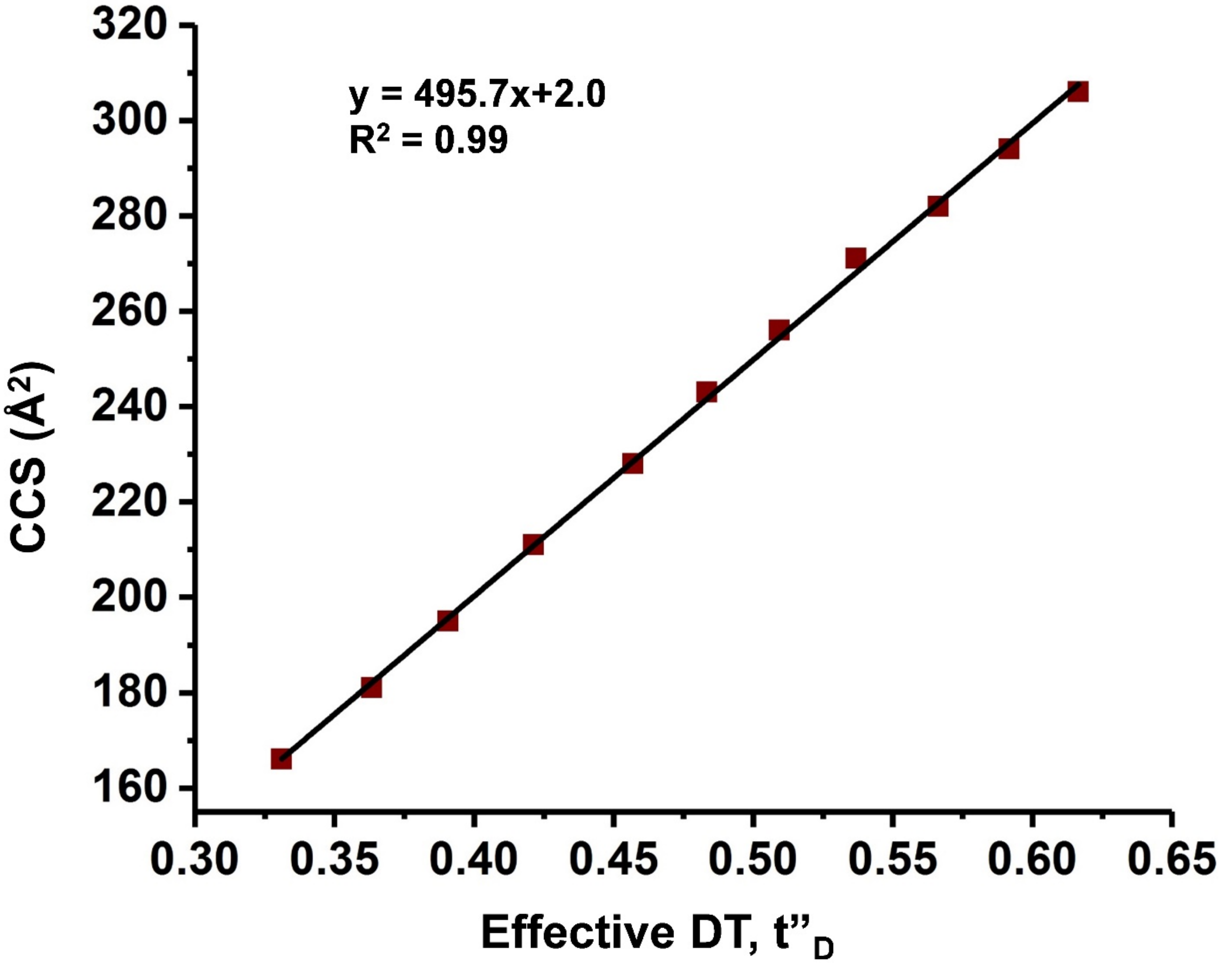

Supplementary Fig. 7. CCS calibration curve based on singly charged polyalanine oligomer mixture (n = 4-14) as the calibrant.

Supplementary Table 1. Neuropeptide ion assignments from cytoplasm of single Type 1 *L. stagnalis* Fgp neuron based on accurate mass measurements and tandem MS by MALDI.

Sequence	Charge State	Formula	m_{meas} (Da)	m_{calc} (Da)	Δm (mDa)	CCS (Å^2)
FLRFa	+1	$[C_{30}H_{44}N_8O_4+H]^+$	581.358	581.356	2	247
FMRFa	+1	$[C_{29}H_{42}N_8O_4S+H]^+$	599.312	599.312	0	246

Supplementary Table 2. Neuropeptide assignments from cytoplasm and nucleus of single *L. stagnalis* Type 2 Fgp neurons based on accurate mass measurements and tandem MS in positive ion mode. Measured CCS values and normalized abundances, I_C, and I_N, for ions detected from cytoplasm and nucleus, respectively, are also shown.

Sequence	Charge State	Formula	m_{meas} (Da)	Δm (mDa)	CCS (Å^2)	I_C (%)	I_N (%)
FLRFa	+1	$[C_{30}H_{44}N_8O_4+H]^+$	581.356[b]	0	247	10.9±3.6	6.0±2.8
GDPFLRFa	+2	$[C_{41}H_{59}N_{11}O_9+2H]^{2+}$	425.740	8	165	11.8±3.7	21.2±8.2
	+2	$[C_{41}H_{59}N_{11}O_9+Na+H]^{2+}$	436.726	3	168		
	+2	$[C_{41}H_{59}N_{11}O_9+K+H]^{2+}$	444.710	0	167		
	+1	$[C_{41}H_{59}N_{11}O_9+H]^+$	850.457[a][b]	0	289		
	+1	$[C_{41}H_{59}N_{11}O_9+Na]^+$	872.438	-1	291		
	+1	$[C_{41}H_{59}N_{11}O_9+K]^+$	888.404	-9	292		
SDPFLRFa	+2	$[C_{42}H_{61}N_{11}O_{10}+2H]^{2+}$	440.744	7	168	15.9±6.1	23.0±6.5
	+2	$[C_{42}H_{61}N_{11}O_{10}+Na+H]^{2+}$	451.730	2	169		
	+2	$[C_{42}H_{61}N_{11}O_{10}+K+H]^{2+}$	459.717	2	169		
	+1	$[C_{42}H_{61}N_{11}O_{10}+H]^+$	880.467[b]	-1	291		
	+1	$[C_{42}H_{61}N_{11}O_{10}+Na]^+$	902.447	-2	295		
	+1	$[C_{42}H_{61}N_{11}O_{10}+K]^+$	918.427	4	291		
SDPYLRFa	+2	$[C_{42}H_{61}N_{11}O_{11}+2H]^{2+}$	448.739	4	168	1.1±0.9	0
	+1	$[C_{42}H_{61}N_{11}O_{11}+H]^+$	896.460[b]	-2	294		
SDPFFRFa	+2	$[C_{45}H_{59}N_{11}O_{10}+2H]^{2+}$	457.734	4	169	0.7±0.3	0
	+1	$[C_{45}H_{59}N_{11}O_{10}+H]^+$	914.446[b]	-6	295		
SKPYMRFa	+2	$[C_{43}H_{66}N_{12}O_9S+2H]^{2+}$	464.254	7	173	4.9±1.9	1.2±1.2
	+2	$[C_{43}H_{66}N_{12}O_9S+Na+H]^{2+}$	475.238	0	175		
	+1	$[C_{43}H_{66}N_{12}O_9S+H]^+$	927.481[b]	-6	297		
	+1	$[C_{43}H_{66}N_{12}O_9S+Na]^+$	949.467	-2	301		
GPSRSSFPRYa	+3	$[C_{51}H_{77}N_{17}O_{14}+3H]^{3+}$	384.873	4	158	2.8±0.9	0
	+2	$[C_{51}H_{77}N_{17}O_{14}+2H]^{2+}$	576.803	4	186		
	+1	$[C_{51}H_{77}N_{17}O_{14}+H]^+$	1152.584[b]	-7	335		
SDPFFRFGKQQVATDDSGELDDEILSRVSDDDKNI	+5	$[C_{169}H_{261}N_{46}O_{64}+5H]^{5+}$	792.578	1	212	1.7±1.2	0.4±0.2
	+4	$[C_{169}H_{261}N_{46}O_{64}+4H]^{4+}$	990.468[a]	-2	222		
	+3	$[C_{169}H_{261}N_{46}O_{64}+3H]^{3+}$	1320.293	2	246		
NNLNNYALEDEDGKLTSDIIDDQFQRYQ	+3	$[C_{142}H_{215}N_{39}O_{54}+3H]^{3+}$	1111.183[a]	0	228	2.7±2.1	3.2±1.9
	+3	$[C_{142}H_{215}N_{39}O_{54}+2H+Na]^{3+}$	1118.513	3	227		
	+3	$[C_{142}H_{215}N_{39}O_{54}+2H+K]^{3+}$	1123.834	-1	227		

[a] Identifications were based on tandem MS by capillary microsampling MS at the single cell level.
[b] Identifications were based on tandem MS on a single *L. stagnalis* visceral ganglion with MALDI-MS.
